# Design and Validation of a Scale to Measure Perceptions and Misconceptions about Menstruation in Nursing College Students: The METCON© Scale

**DOI:** 10.3390/healthcare12181836

**Published:** 2024-09-13

**Authors:** Alicia Botello-Hermosa, Silvia Escribano-Cubas, María Dolores Guerra-Martín, Nicia del Rocío Santana-Berlanga, Rocío Cáceres-Matos

**Affiliations:** 1 Research Group SEJ066, Women, Wellbeing and Citizens, 41930 Bormujos, Spain; abotello@us.es; 2 Nursing Department, Faculty of Nursing, Physiotherapy and Podiatry, University of Seville, 6 Avenzoar ST, 41009 Seville, Spain; nsantana@us.es; 3 Nursing Department, Faculty of Health Sciences, Alicante University, San Vicente del Raspeig Road, 03690 Alicante, Spain; silvia.escribano@ua.es; 4 Institute of Biomedicine of Seville (IBiS), Antonio Maura Montaner ST, 41013 Seville, Spain; 5 Research Group CTS969, Care Innovation and Health Determinants, 41009 Seville, Spain; 6 Research Group CTS-1141, Clinical Research Applied to Care and New Care Paradigms, 41009 Seville, Spain; 7 Emergency Nurse, San Juan de Dios Aljarafe Hospital, San Juan de Dios Av., Bormujos, 41913 Seville, Spain; 8 Research Group PAIDI-CTS-1050, Complex Care, Chronicity and Health Outcomes, 41009 Seville, Spain

**Keywords:** gender stereotypes, menstruation, nursing students, perceptions, psychometrics, structural validity, taboos

## Abstract

Menstruation remains surrounded by taboo and symbolic violence for many adolescents and young women today, and it is urgent to identify existing stereotypes. The objective was to validate a Spanish-language instrument to assess perceptions, false beliefs, gender stereotypes, fears, and taboos surrounding menstruation in nursing women’s studies. A cross-sectional study for psychometric validation of the METCON© scale (22 item version) was assessed in a cohort of 401 female nursing university students (2016–2019), assessing the psychometric properties in terms of internal consistency and structural validity. Furthermore, an invariance analysis was conducted to discern structural disparities before and after studying the subject of gender and health. The METCON© scale showed acceptable internal consistency scores measured by Cronbach’s alpha. Exploratory factor analysis indicated a structure of six factors which explained 60.50% of the total variance with a total of 19 items. Reliability analysis indicated adequate internal consistency (α = 0.613). Confirmatory factor analysis showed adequate values, confirming this structure. The invariance analyses identified that the structure remained adequate when evaluated before and after studying the subject of gender and health. Once validated, this instrument could serve as a valid and reliable tool for comprehensively examining perceptions, beliefs, and myths surrounding menstruation, addressing not only biological but also social and cultural aspects.

## 1. Introduction

More than 30 million women are menstruating every day [1]. However, menstruation remains surrounded by taboo and symbolic violence for many adolescents and young women today [2,3], which manifests in the form of concealment and the silence of both women and men on the subject [4].

Negative societal comments related to the perceived uncleanliness of menstruation channel feelings of insecurity and shame, leading to silence [1,5,6] and effectively making people complicit in their own victimisation [7]. This concealment is observed in popular language through the abundance of metaphors used to refer to the menstrual cycle [8].

Worldwide, it is estimated that there may be approximately 60 billion euphemisms related to the menstrual cycle [9,10]. Although it cannot be asserted that the menstrual cycle remains as taboo a topic in Spain, there is a clear overall lack of knowledge about it. Additionally, popular imagination, through culture, has perpetuated myths about women’s reproductive systems, particularly menstruation, which still persist in gender stereotypes and biases [11].

In this regard, it is urgent to review how the fundamental stages of women’s lives have been defined, what stereotypes exist, what they have represented for the women themselves, and how they have affected their health [12], all with the purpose of identifying them and proposing strategies for correction [8]. According to Martínez, Parera, and Rius, for appropriate management of menstruation, it is necessary to understand how women experience it and how it affects and limits them, which are factors influenced by the sociocultural environment. Information about these aspects in Spanish women is scarce [13]. This comprehensive approach (biological, cultural, and social) is necessary for correct and effective management of menstruation [14].

The METCON© scale was preliminarily designed and piloted by Botello-Hermosa in 2016 [15] in Spain with the objective of assessing knowledge, myths, and gender stereotypes regarding menstruation and the menstrual cycle. However, it is necessary to proceed with the validation process by confirming its factorial structure. Therefore, the present study has the objective of validating a Spanish-language instrument to assess perceptions, false beliefs, gender stereotypes, fears, and taboos surrounding menstruation in college-aged young women.

## 2. Materials and Methods

### 2.1. Design and Sample Size

A cross-sectional validation study with non-probability sampling for convenience was used to test the internal consistency and structure of the METCON© scale. The Sex and Gender Equity in Research (SAGER) guidelines and the SAGER guidelines checklist were followed in this manuscript. The sample size calculation took into account the recommendations of McCallum [16] and the COnsensus-based Standards for the selection of health Measurement INstruments Checklist Statement (COSMIN Checklist Statement) [17], which estimate that a minimum of 5 and 7 individuals per item require at least 110 and 154 subjects, respectively.

The sample size to be used was calculated using G*Power V.3.1.9.4. The five input parameters were two-tailed, with a large effect size (f^2^ = 0.35), a power of 0.95, a statistical significance of 95% (α = 0.05), and a total of 22 predictors [18,19,20]. The minimum number of subjects was considered to be 137.

### 2.2. Data Collection

The participants were female students in the first and second year of degrees in nursing at the Faculty of Nursing, Physiotherapy and Podiatry of the University of Seville. All participants were women since the scale focuses on first-person experiences about menstruation in the participants’ lives.

Participants were recruited during the month of December for the 2016–2017, 2017–2018, and 2018–2019 academic years. A total of 401 nursing students agreed to participate out of the 734 eligible. All of them completed the scale individually and during class hours with prior authorisation from the professors involved. Response times ranged from 8 to 12 min. The inclusion criteria were female students over the age of 18 enrolled in the first or second year for degrees in nursing.

The following sociodemographic data were collected: age, grade (first or second year) and place of residence (rural or urban, categorised through a specific question about the place of residence). The data on the population’s perception of menstruation using the METCON© scale were also collected.

### 2.3. Instrument

The METCON© scale was designed and piloted by Botello-Hermosa et al. [15] with the objective of evaluating the perceptions that adolescent and young women have about menstruation and the menstrual cycle, specifically myths, stereotypes and knowledge (Supplementary File S1). The scale assesses the perception of menstruation from a comprehensive perspective (biological, cultural and social), which is necessary for correct management of it.

In the preliminary piloting study, the 22 item METCON© scale showed adequate internal consistency through the calculation of Cronbach’s alpha coefficient (α = 0.724). The exploratory factor analysis (EFA) indicated that it is structured into four dimensions (67.98% of the explained variance) which make up the theoretical construct of perception of menstruation. The four dimensions were (1) menstruation as a taboo subject, (2) gender stereotypes related to menstruation and menopause, (3) false beliefs and myths about menstruation, as well as fears and prohibitions related to menstruation, and (4) knowledge about menstruation.

Each item is assessed using a Likert scale with five response options ranging from 1 (strongly disagree) to 5 (strongly agree). Consequently, a higher score indicates a more negative perception of menstruation. Additionally, the positive and negative implications of each item were considered. Specifically, items 4, 19, 20 and 21 were negatively worded, and their scores were reversed. In these cases, the item values ranged from 1, representing the least favorable situation, to 5, representing the most favorable situation.

### 2.4. Ethical Aspects

Detailed information about the study, namely the expressed request for informed consent, the voluntary nature of participation as well as the treatment of the data, were included in the questionnaire, informing the participants that they could withdraw at any time.

The Declaration of Helsinki on ethical principles for research with human beings was followed throughout the process, and this project had the approval of and favorable opinion from the Research Ethics Committee of the Virgen Macarena and Virgen del Rocío university hospitals (record 14/2016 (22 December 2016)).

### 2.5. Data Analyses

Descriptive statistics were employed to map and summarise the characteristics of the sample. Continuous variables were reported as mean values ($\bar{x}$) with a confidence interval (CI), while categorical variables were presented as percentages (%) along with their respective CIs. Differences in proportions among groups were assessed using the χ^2^ test for categorical variables and the Student’s *t*-test for continuous variables. A significance level of 0.05 was adopted, and normality was assessed using the Kolmogorov–Smirnov test.

Firstly, the positive and negative meaning of each item was considered. The values of items 4 and 19–21 were rotated due to their negative meanings (the least favourable situation) to five (the most favourable situation).

To carry out the factorial analyses, the sample was divided into two randomised subsamples to perform EFA (n = 200) and confirmatory factor analysis (CFA) (n = 201) [21]. To check the structure of the scale, EFA was performed using the extraction methods of robust maximum likelihood estimation to mitigate the possible biases which could occur in the estimates due to the observed floor effects [22].

Three required assumptions were verified: (1) the scale scores were confirmed to have a normal distribution; (2) the items demonstrated at least moderate correlations with one another, with corrected item-total correlations exceeding 0.4, indicating a moderate effect; and (3) Bartlett’s sphericity index was calculated with a significance level of *p* < 0.05, and the Kaiser–Meyer–Olkin (KMO) measure was assessed to be higher than 0.8. For the extraction of the components, factors with eigenvalues >1 were retained, with a cross-loading saturation ≥0.30 [23,24]. An explained variance of more than 60% was required, as well as content validity with the factor [25]. The number of suitable factors was made by parallel analysis [26].

Internal consistency was assessed using Cronbach’s alpha, which ranged from 0 to 1. Values of Cronbach’s alpha between 0.7 and 0.8 indicate acceptable internal consistency, values between 0.8 and 0.9 reflect good consistency, and values greater than 0.9 denote excellent internal consistency [27]. The skewness, kurtosis, and floor and ceiling effects were also evaluated and considered adequate when less than 15% of the subjects in the lower and upper ranges responded to the extreme values, both for individual items and subscales [28,29].

CFA was conducted, and the acceptability of the factor solutions was evaluated based on the goodness of fit index (GFI > 0.90), the standardised root mean residuals (SRMR < 0.08), and the root mean squared error of approximation (RMSEA < 0.10) [30].

To determine the threshold values for both the overall scale and its respective subscales, we employed distinct methodologies contingent on whether the data adhered to a normal distribution pattern. When the data exhibited a normal distribution, we applied the effect size metric. Cohen suggests that the initial threshold corresponds to an effect size of 0.5 (mean effect size) and the 69th percentile. The second threshold aligns with the 79th percentile, equivalent to an effect size of 0.8, which is characterised as substantial [31]. In cases where the data deviated from a normal distribution, we employed a methodology based on the item’s discriminatory power. This involved calculating the item’s discriminant index and the adjustment factor while considering the weights of each item within the entire scale [32,33,34,35].

Invariant analysis was conducted through multigroup confirmatory factor analysis (MGCFA), employing an unconstrained-constrained approach. Initially, an unconstrained model was utilised, permitting the parameters to vary freely. This model, also known as a configural model, maintains identical items for factors in both groups and serves as a baseline for subsequent model evaluations. Following this, a fully constrained model was implemented, where the parameters were restricted to be consistent across groups (gender: women and men), evaluating metric and scalar invariance. Metric invariance examines whether the factor loadings are equivalent across groups, ensuring that the underlying meaning of the common factors remains consistent across these groups. Scalar invariance, on the other hand, ensures that the item intercepts are equivalent across different groups, indicating that they are not influenced by external factors or group-specific attributes. Furthermore, scalar invariance implies that the means of the latent variables are comparable across groups [36].

The assumption of structural invariance is supported if the MGCFA meets the following criteria [37]:

(1) The model, which specifies the items measuring each latent variable, fits the data well. Several fit indices were evaluated to test the confirmatory factor analysis (CFA) structure of the model: RMSEA < 0.08 [38,39]; CFI > 0.90 [40,41]; the χ^2^ test (a non-significant χ^2^ test (*p* value > 0.05) indicates an acceptable model structure); and the ratio of χ^2^ to degrees of freedom (df) (χ^2^/df < 3) [38,41].
(2) All factor loadings are substantial (exceeding 0.30) and statistically significant.
(3) There are no significant modification indices suggesting model misspecifications. Model fit was assessed using an χ^2^ difference test (χ^2^_diff_), considering the change in degrees of freedom between models with a cutoff of 0.01 [36,42]. Additionally, differences in CFI (ΔCFI) and RMSEA (ΔRMSEA) of less than 0.01 and 0.015, respectively, were required between the unconstrained and constrained models.

All statistical analyses were conducted using version 2.7.2 of the free software R (The R Project, Auckland, New Zealand) and IBM SPSS^®^ version 26.0 (IBM Corp., Armonk, NY, USA), and structural analyses were performed using Analysis of Moment Structures (AMOS©) version 26 software (IBM Corp., Armonk, NY, USA).

## 3. Results

### 3.1. Characteristics of the Participants and Descriptive Analysis of the Items of the 22 Item METCON© Scale

This study’s subjects represented a sample of 401 women with an average age of 20.36 years (SD = 4.37) from the first (32.4%) and second (67.6%) years of studies for degrees in nursing (first year (before studying the subject gender and health) = 130; second year (after studying the subject gender and health) = 271). A total of 59.4% lived in an urban environment, and 40.6% lived in a rural environment. Regarding religion, 72.1% were Catholic (Protestant = 0.7%; Muslim = 0.2%; other = 22.1%).

Regarding the items, the distribution of 7 items (36.84%) for the 22-item scale did not follow the normality of the data, with absolute values above ±1.5 for skewness and kurtosis. Table 1 shows the mean values, standard deviation and range of values for each item as well as other descriptive data.

### 3.2. Exploratory Factor Analysis and Internal Consistency of the METCON© Scale

Through EFA, it was verified that the distribution of the data followed a normal distribution (*p* < 0.001) in line with the assumption of correlation between variables. Table 1 presents the corrected item-total correlation values, which exceeded the recommended minimum value of 0.3. The results of the EFA with the 22 items revealed an initial structure of six factors, with a KMO of 0.86, a correlation matrix determinant of 0.048, and a Bartlett’s sphericity test result of 1190.4392 (df = 1231; *p* < 0.001), indicating that the EFA was feasible.

Based on this EFA and conceptual previous research, a six-factor solution appeared to be best, according to the test score and eigenvalues. Table 2 displays the cross-loadings for each item on the different factor solutions. Items 9, 19 and 22 showed cross-loadings <0.4 for every factor. Therefore, the 19 remaining items were grouped into six factors with an explanatory power of variance of 60.50%.

Table 1 also shows information regarding the ceiling and floor effects on any individual item above 15%. Items 2, 3 and 5 showed a ceiling effect, and the majority (89.47%) had a floor effect.

The internal consistency of the total scale, comprising 22 items, yielded a Cronbach’s alpha coefficient of 0.602. Cronbach’s alpha was also recalculated upon the removal of each item (see Table 1). Removing items 9, 19 and 22 increased the Cronbach’s alpha value of the overall scale to 0.613. Therefore, a total of 19 items were proposed to be included in the CFA.

### 3.3. Confirmatory Factor Analysis Subscales

The results of the CFA showed fit indices excellent for the proposed six-factor subscales model (Chi square (χ^2^ = 244.93; *p* < 0.001); CFI = 0.902; TLI = 953; NFI = 0.965; RMSEA = 0.063 (90% CI: 0.050–0.076)).

Floor or ceiling effects were not found for either subscale above 15% (Table 2), obtaining good values for internal consistency for all subscales (>0.70).

**Figure 1:**
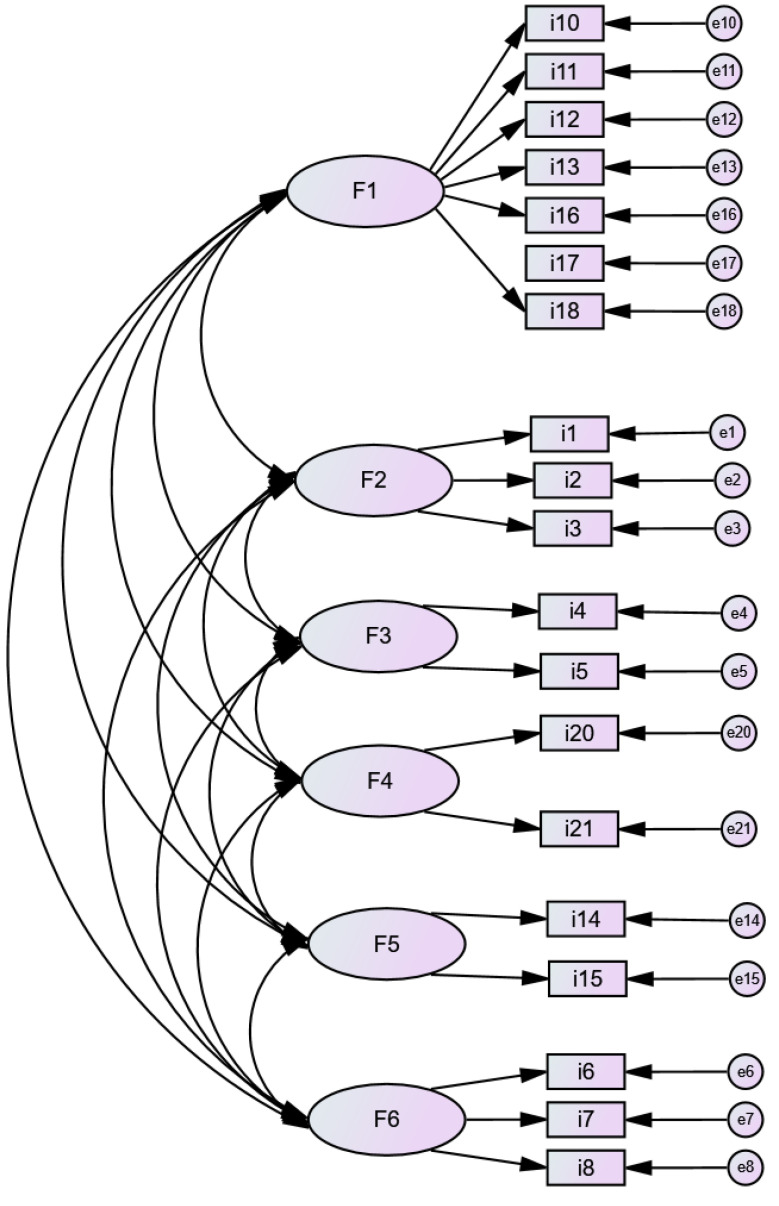
Path diagram analysis.

Additionally, the naming of each construct within the scale was based on the previously established theoretical assumptions as follows: factor 1 is for fears and prohibitions about menstruation (seven items); factor 2 is for menstrual taboos relating to environments (three items); factor 3 is for experiences about menstrual taboos (two items); factor 4 is for knowledge (two items); factor 5 is for false beliefs (two items), and factor 6 is for gender stereotypes (three items). Figure 1 shows the diagram of the structure of the scale.

### 3.4. Cut-Off Points

Since neither the subscale scores nor the overall scale followed a normal distribution, the procedure was based on the discriminative power of each item. This involved calculating each item’s discriminant index and the correction factor, taking into account the weight of each item within the entire scale. The cut-off points are detailed in Table 3.

### 3.5. Evaluation of the Invariance of the Measurement Model

The results of the MGCFA and χ^2^_diff_ test indicated that there was moderation based on the academic course at the model level. Initially, the unconstrained model for the six-factor solution was evaluated. The fit indices demonstrated that the model adequately represented the data (CFI = 0.904; RMSEA = 0.057, 95% CI (0.048–0.067); χ^2^/df = 1.65) for comparing invariance with the metric and scalar models (Table 4). Additionally, all standardised factor loadings for this six-factor model exceeded 0.30 (Table 3).

Table 4 presents a comparison of the fit indicators among the various calculated invariance models. First, the metric invariance model was examined, wherein the factor loadings were constrained to be equal between both courses. The indices revealed that the model fit well, but in comparison with the unconstrained model, χ^2^_diff_ was significant (*p* < 0.001). Nevertheless, the ΔRMSEA was less than 0.015, and the ΔCFI was below 0.01. These results indicate that the model did not demonstrate metric invariance.

The evaluation of the scalar invariance model, where both intercepts and factor loadings were constrained to be equal across groups (by course), showed a good fit. However, when compared with the metric invariance model, significant differences were found in χ^2^_diff_ (*p* < 0.001). In contrast, no notable changes were observed in the RMSEA (ΔRMSEA < 0.015) or in the CFI (ΔCFI < 0.01). These findings suggest that the model does not possess scalar invariance.

Finally, the strict invariance model, which imposed equal constraints on error variances as well as factor loadings and intercepts, also demonstrated a good fit. However, upon comparison with the scalar invariance model, significant changes were noted in χ^2^_diff_ (*p* < 0.001). Conversely, no significant changes were observed in the RMSEA (ΔRMSEA < 0.015) or the CFI (ΔCFI < 0.01). These results signify that the model does not demonstrate strict invariance.

In this regard, it is worth noting that since the scales were administered in December, second-year students had already completed the “Gender and Health” course, which is taught in the second semester of the first year (the previous academic year). Additionally, in their second year, they were in the final stages of their instruction in the courses “Nursing in Reproductive Health” and “Nursing for Infants and Adolescents”, both of which are offered in the first semester.

## 4. Discussion

The present study constitutes the initial psychometric evaluation of the METCON© scale [15], designed for appraising perceptions, misconceptions, gender stereotypes, anxieties, and cultural taboos pertaining to menstruation in college-aged females. A 19 item instrument with six distinct factors was derived based on its psychometric properties and structural analysis.

In terms of internal consistency, the instrument demonstrated an acceptable score, with a Cronbach’s alpha of 0.613. It is important to note that during the initial stages of validation, a Cronbach’s alpha value near 0.60 is considered acceptable [43,44,45], indicating that further validation is necessary. Generally, as Cronbach’s alpha approaches one, internal consistency improves [46]. However, it is also noted that values exceeding 0.9 might suggest redundancy in items or constructs [47]. In this study, all factors showed adequate internal consistency, with values ranging between 0.60 and 0.84 [48].

Additionally, the results revealed that most items exhibited floor effects [28], suggesting that these items did not adequately capture variability in responses. To address this issue, the confirmatory factor analysis (CFA) was conducted using the robust maximum likelihood estimation model to reduce potential biases [22]. The low scores on various items may be attributed to the fact that the participating students were enrolled in a higher-education setting within the health sciences field and had specific training at their institution.

In this context, it is important to highlight that, as the scales were administered in December, second-year students had already completed the “Gender and Health” course, which is offered during the second semester of the first year (i.e., the previous academic year). Furthermore, in their second year, they were nearing completion of the courses “Nursing in Reproductive Health” and “Nursing for Infants and Adolescents”, both of which are taught in the first semester. These three courses explore menstruation and the menstrual cycle from various social, cultural and biological perspectives [49], contributing to higher levels of knowledge, as evidenced by the results. Conversely, first-year students, at the time of data collection (first semester), had not yet taken any of these three courses.

Furthermore, in the factorial structure, it was observed that three out of the six dimensions consisted of only two items, whereas Nunnaly recommends a minimum of three [44,45,46]. This could have introduced a bias in the results. Therefore, it is advised to reevaluate the scale’s structure to determine if it remains invariant when considering specific groups or particular characteristics. In line with the aforementioned factors, regarding students who had completed various related courses, the scale’s invariance was assessed by considering whether the participating students were in their first or second year.

The results indicated that the instrument remained stable when evaluated separately for the two groups—first-year and second-year students—although it differed between the two groups. This disparity may be attributed to the distinct academic training received in each year. This suggests that there might be items with a differential understanding, warranting an evaluation in a future study to ascertain if second-year students hold fewer misconceptions about menstruation. This methodology, invariance analyses using MGCFA, represents an advanced psychometric analysis approach which allows for the identification of whether a construct holds a different meaning in distinct groups or sub-samples due to their particular characteristics [50]. However, despite the adequacy of the structures, it would be prudent to continue refining the items comprising the scale, considering the addition of new items to achieve a minimum of three items per subscale.

Regarding the scale’s dimensions, those showing less favorable scores in relation to the scale’s objectives were the subscales labeled “knowledge” and “taboo”. These two subscales may be interrelated, as it appears that young women seek more information and knowledge due to the continued presence of menstrual taboos among participants, according to the obtained results. Thus, a higher degree of taboo corresponds to a greater lack of information or knowledge. This is a noteworthy finding, particularly considering the participants were nursing university students. The results suggest that menstrual taboos persist, potentially leading to inadequate depth and quality of discussion both within educational settings and households. Furthermore, menstrual taboos are linked to insufficient knowledge, prompting many girls and adolescents to seek information from their peer groups, often resulting in inaccurate and stereotypical information [51]. This finding aligns with our results, indicating that peer groups serve as a more comfortable and significant source of information.

As for future directions, it would be beneficial to explore disciplines outside of health sciences and across different academic levels for comparative purposes. Additionally, it should be assessed whether there are differences in perceptions and beliefs before and after receiving menstrual health education. Furthermore, it may be worthwhile to consider other environments where distinctions may arise, such as urban versus rural settings. This approach also allows for an evaluation of the quality of menstrual information received by participants throughout their academic training.

Understanding and addressing misconceptions about menstruation is crucial for designing effective public policies in both societal and educational spheres. By identifying and correcting these misconceptions, policymakers can promote comprehensive education about menstruation in schools, thereby reducing the stigma and fostering a better understanding of the biological and emotional aspects related to this natural process. Additionally, addressing misconceptions can contribute to promoting gender equity by challenging stereotypes and discrimination based on gender. This, in turn, can lead to improved access to menstrual hygiene products and healthcare services, particularly for marginalised communities. Overall, tackling misconceptions about menstruation can lead to more inclusive policies which support education, health, gender equity and access to essential resources for all menstruators.

Finally, this study presented a series of strengths and limitations. Among the strengths, we would highlight its exploration of a topic of both interest and novelty, being particularly relevant in the field of nursing. Firstly, it is worth noting that due to social desirability, participating students may have been inclined to provide responses indicating a more favorable perception across various items. Secondly, possibly related to the aforementioned limitation, given the presence of floor effects, a CFA was conducted employing the robust maximum-likelihood estimation model to mitigate potential biases. Additionally, although the percentage of participants from other nationalities or cultures was quite small, it was not considered a factor which could potentially influence the understanding of the items. On the other hand, we consider it necessary to test this instrument on a general population.

## 5. Conclusions

The 19 item METCON© scale demonstrated satisfactory internal consistency and a robust factorial structure, confirmed through confirmatory factor analysis, when delineating six dimensions. Following validation, this instrument holds the potential to serve as a valid and reliable tool for comprehensively examining perceptions, beliefs and myths surrounding menstruation. It not only encompasses biological aspects but also addresses the social and cultural dimensions of this phenomenon. In future studies, we consider it necessary to validate the instrument in a general population and not just among women. Additionally, it would be interesting to assess the instrument’s validity in men.

## Figures and Tables

**Table 1:** Descriptive data of the items and reliability analysis of the METCON© scale (22 items) (n = 401).

	Mean (SD) *	Skewness	Kurtosis	Scale Mean if Item Removed	Scale Variance if Item Removed	Corrected Item-Total Correlation	Cronbach’s Alpha if Item Removed	Floor Effect ** n (%)	Ceiling Effect *** n (%)
1. At my school or institute, menstruation was a more taboo subject than at home.	3.02 (1.28)	−0.13	−1.04	42.44	53.12	0.57	0.600	65 (16.2)	54 (13.5)
2. Among friends, cousins or sisters, menstruation was a less taboo topic than at home.	3.14 (1.32)	−0.14	−1.10	42.35	51.17	0.69	0.587	57 (14.2)	76 (19.0)
3. Among friends, cousins or sisters, menstruation was a less taboo topic than at my school or institute.	3.62 (1.27)	−0.77	−0.42	41.87	51.45	0.54	0.587	42 (10.5)	113 (28.2)
4. When I first had my period, I knew what it was and I felt safe.	2.73 (1.26)	0.19	−1.06	42.77	51.34	0.40	0.585	79 (19.7)	36 (9.0)
5. I was scared when I first saw menstrual blood.	2.76 (1.47)	0.20	−1.40	42.74	48.74	0.31	0.572	115 (28.7)	66 (16.5)
6. During menstruation, you felt sick or become sick.	2.12 (1.26)	0.87	−0.37	43.34	50.04	0.33	0.571	176 (43.9)	27 (6.7)
7. Menopause means a woman ages because she no longer has menstruation.	2.04 (1.08)	0.88	−0.01	43.43	51.52	0.30	0.576	156 (38.9)	11 (2.7)
8. When you reach menopause, you lose sexual attractiveness.	1.51 (0.84)	1.82	3.16	43.96	54.04	0.41	0.589	266 (66.3)	4 (1.0)
9. During menopause, medical treatment is required (anxiety, depression, hot flashes...).	2.41 (1.09)	0.348	−0.58	43.05	53.31	0.20	0.613	13 (3.2)	2 (0.5)
10. Washing your head during menstruation can be dangerous for your health.	1.10 (0.42)	4.76	24.02	44.36	55.98	0.35	0.595	375 (93.5)	3 (0.7)
11. If I prepare food like mayonnaise during menstruation, it spoils or separates easily.	1.13 (0.53)	4.81	25.12	44.33	55.96	0.52	0.593	370 (92.3)	2 (0.5)
12. During menstruation, you should not bathe in the sea or swimming pools.	1.21 (0.69)	3.90	15.68	44.25	54.75	0.55	0.591	356 (88.8)	6 (1.5)
13. Menstrual blood is toxic, so it can have harmful effects on foods or drinks that are touched during menstruation.	1.13 (0.47)	4.89	29.08	44.33	56.65	0.35	0.601	365 (91.0)	2 (0.5)
14. Menstrual blood is an accumulation of dirt and waste.	1.59 (0.95)	1.54	1.32	43.87	52.34	0.31	0.578	263 (65.5)	2 (0.5)
15. Menstrual blood has an unpleasant decomposing odor.	2.75 (1.21)	0.03	−0.99	42.74	50.84	0.59	0.577	81 (20.2)	27 (6.7)
16. Menstrual blood is incompatible with water.	1.26 (0.70)	3.25	11.18	44.20	54.57	0.60	0.589	337 (84)	4 (1.0)
17. During menstruation, you should not have sexual relations.	1.71 (0.99)	1.36	1.18	43.75	53.71	0.35	0.592	231 (57.6)	8 (2.0)
18. During menstruation, you should not shower because you cut yourself and it is dangerous for your health.	1.17 (0.64)	4.58	22.22	44.29	55.24	0.39	0.594	365 (91.0)	7 (1.7)
19. There were people around me (family, friends, at school) who told me what menstruation was.	2.09 (1.24)	1.13	0.32	43.37	53.22	0.25	0.609	35 (8.7)	44 (11.0)
20. I know my menstrual cycle and what happens in my body.	1.65 (0.91)	1.71	3.10	43.81	54.44	0.46	0.595	221 (55.1)	9 (2.2)
21. I know that when in the menstrual cycle, I am most fertile.	2.16 (1.17)	0.92	−0.02	43.30	54.88	0.51	0.598	138 (34.4)	22 (5.5)
22. I need to receive more information about the menstrual cycle.	3.33 (1.18)	−0.26	−0.79	42.13	56.01	0.21	0.625	74 (18.5)	113 (28.2)

* 0% = missing value. ** Percent score for best possible value. *** Percent score for worst possible value. Note: Items 4, 19, 20 and 21 are rotated.

**Table 2:** Rotated component matrix: METCON© scale (22 items).

	Factors					
	Factor 1	Factor 2	Factor 3	Factor 4	Factor 5	Factor 6
Cumulative Proportion of Variance Explained	13.40%	23.90%	32.302%	46.50%	52.40%	60.50%
1. At my school or institute, menstruation was a more taboo subject than at home.		0.54				
2. Among friends, cousins or sisters, menstruation was a less taboo topic than at home.		0.52				
3. Among friends, cousins or sisters, menstruation was a less taboo topic than at my school or institute.		0.93				
4. When I first had my period, I knew what it was and I felt safe.			0.77			
5. I was scared when I first saw menstrual blood.			0.67			
6. During menstruation, you feel sick or become sick.						0.40
7. Menopause means a woman ages because she no longer has menstruation.						0.53
8. When you reach menopause, you lose sexual attractiveness.						0.57
9. During menopause, medical treatment is required (anxiety, depression, hot flashes...). *						
10. Washing your head during menstruation can be dangerous for your health.	0.54					
11. If I prepare food like mayonnaise during menstruation, it spoils or separates easily.	0.36					
12. During menstruation, you should not bathe in the sea or swimming pools.	0.66					
13. Menstrual blood is toxic, so it can have harmful effects on foods or drinks that are touched during menstruation.	0.36					
14. Menstrual blood is an accumulation of dirt and waste.					0.71	
15. Menstrual blood has an unpleasant decomposing odor.					0.52	
16. Menstrual blood is incompatible with water.	0.32					
17. During menstruation, you should not have sexual relations.	0.38					
18. During menstruation, you should not shower because you cut yourself and it is dangerous for your health.	0.55					
19. There were people around me (family, friends, at school) who told me what menstruation was. *						
20. I know my menstrual cycle and what happens in my body.				0.90		
21. I know that when in the menstrual cycle, I am most fertile.				0.49		
22. I need to receive more information about the menstrual cycle *						

Extraction method: principal component analysis. Rotation method: Varimax with Kaiser normalisation. Loadings <0.40 were removed. * Items with cross-loadings <0.4 for each factor solution.

**Table 3:** Internal consistency of each subscale *.

Subscale	Mean *	SD	Minimum	Maximum	Cut-Off Points	Kolmogorov–Smirnov	% Floor Effect **	% Ceiling Effect ***	Cronbach’s Alpha
Factor 1	8.71	2.54	7	35	8–21	0.702 ****	0.8	2.3	0.84
Factor 2	9.73	3.08	3	15	10–13	0.961 ****	8.6	1.0	0.71
Factor 3	5.41	2.43	2	10	6–8	0.952 ****	1.8	6.3	0.72
Factor 4	3.81	1.76	2	10	3–6	0.872 ****	13.4	7.6	0.72
Factor 5	4.31	1.84	2	10	3–7	0.935 ****	6.1	0.3	0.67
Factor 6	5.67	2.27	3	15	6–8	0.916 ****	1.0	3.3	0.60
Scale	62.00	6.88	19	95	33–49	0.987 ****	0.3	0.3	0.61

SD = standard deviation. * 0% indicates missing value. ** Percent scoring of worst possible value. *** Percent scoring of best possible value. **** Indicates *p* value < 0.001.

**Table 4:** Comparison of the fit index between invariance models.

Model	χ^2^	χ^2^/(df)	CFI	RMSEA (95% CI)	χ^2^_diff_ (df); *p* Value	ΔCFI	ΔRMSEA
Unconstrained							
Metric invariance model	474.65	1.65	0.904	0.057 (0.048–0.067)	45.68 (13); *p* < 0.001	−0.052	0.004
Scalar invariance model	505.61	1.65	0.985	0.057 (0.048–0.066)	30.96 (19); *p* < 0.001	−0.003	0.000
Strict invariance model	588.55	1.80	0.987	0.040 (0.037–0.043)	82.94 (21); *p* < 0.001	−0.098	0.007

χ^2^ = Chi square test; χ^2^_diff_ = χ^2^ difference test; CFI = comparative fit index; CI = confidence interval; df = degrees of freedom; RMSEA = root-mean squared error of approximation.

## Data Availability

Data are contained within the article and Supplementary Materials.

## Supplementary Materials

The following supporting information can be downloaded at: https://www.mdpi.com/article/10.3390/healthcare12181836/s1, File S1. METCON© Instrument.

## References

[1] Hennegan J., Shannon A.K., Rubli J., Schwab K.J., Melendez-Torres G.J. (2019). Women’s and girls’ experiences of menstruation in low- and middle-income countries: A systematic review and qualitative metasynthesis. PLoS Med..

[2] Feijóo-Tituana M.B. (2016). Tapua la Menstruación Como Parte de los Ciclos de la Violencia Simbólica. Doctoral Dissertation.

[3] Shah V., Nabwera H.M., Sosseh F., Jallow Y., Comma E., Keita O., Torondel B. (2019). A rite of passage: A mixed methodology study about knowledge, perceptions and practices of menstrual hygiene management in rural Gambia. BMC Public Health.

[4] Valls-Llobet C. (2010). Women, Health and Power.

[5] Borjigen A., Huang C., Liu M., Lu J., Peng H., Sapkota C., Sheng J. (2019). Status and Factors of Menstrual Knowledge, Attitudes, Behaviors and Their Correlation with Psychological Stress in Adolescent Girls. J. Pediatr. Adolesc. Gynecol..

[6] Newton V.L. (2016). Everyday Discourses of Menstruation: Cultural and Social Perspectives.

[7] Bourdieu P. (2021). Symbolic Violence. Lat. Sociol. J..

[8] Botello-Hermosa A., Casado-Mejía R. (2017). Cultural Meaning of Menstruation in Spanish Women. Sci. Nurs..

[9] Stein E., Flow S.K. (2009). The Cultural Study of Menstruation.

[10] Thiébaut E. (2018). This is my blood. Little History of Menstruation(s), of Those Who Have Them and of Those Who Mark Them.

[11] García-Dauder S., Pérez-Sedeño E. (2018). The “Lies” about Women in Science.

[12] Chandra-Mouli V., Patel S.V. (2017). Mapping the knowledge and understanding of menarche, menstrual hygiene and menstrual health among adolescent girls in low- and middle-income countries. Reprod. Health.

[13] Martínez-San Andrés F., Parera-Junyent N., Rius-Tarruella J. (2018). Characteristics and Impact of Menstruation in Spanish Women: Reasons for Interest in Menstrual Suppression. Reprod. Med. Clin. Embryol..

[14] Chew K.S., Wong SS L., Hassan A.K., Po K.E., Zulkhairi N., Yusman N.A.L. (2021). Development of a validated instrument on socio-cultural and religious influences during menstruation in Malaysia. Med. J. Malays..

[15] Botello-Hermosa A., García-Jiménez M., Santana-Berlanga N.R., Ruiz-Ferrón C. (2019). Design and Validation of a Questionnaire to Measure the Knowledge and Attitudes of Young Women towards Menstruation: Metcon Scale (Botello-Hermosa 2018). Feminismo/s.

[16] McCallum A. (1993). What is an outcome and why look at them?. Crit. Public Health.

[17] Mokkink L.B., Terwee C.B., Patrick D.L., Alonso J., Stratford P.W., Knol D.L., de Vet H.C.W. (2012). The COSMIN Checklist Manual. Qual. Life Res..

[18] Cohen J. (1988). Statistical Power Analysis for the Behavioral Sciences.

[19] Faul F., Erdfelder E., Lang A.G., Buchner A. (2007). G*Power 3: A flexible statistical power analysis program for the social, behavioral, and biomedical sciences. Behav. Res. Method.

[20] Green B.G. (1991). How many subjects does it take to do a regression analysis?. Multiv. Behav. Res..

[21] Lloret-Segura S., Ferreres-Traver A., Hernández-Baeza A., Tomás-Marco I. (2014). Exploratory factor analysis of items: A practical guide, revised and updated. An. Psicol..

[22] Brown T.A. (2015). Confirmatory Factor Analysis for Applied Research.

[23] Tabachnick B., Fidell L. (2013). Using Multivariate Statistics.

[24] Bandalos D.L., Finney S.J., Hancock G.R., Mueller R.O. (2010). Factor Analysis: Exploratory and Confirmatory. Reviewer’s Guide to Quantitative Methods.

[25] Bartlett M.S. (1950). Tests of Significance in Factor Analysis. Br. J. Mathemat. Stat. Psychol..

[26] Horn J.L. (1965). A rationale and test for the number of factors in a factor analysis. Psychometrika.

[27] Cronbach L.J. (1951). Coefficient alpha and the internal structure of tests. Psychometrika.

[28] Lim C.R., Harris K., Dawson J., Beard D.J., Fitzpatrick R., Price A.J. (2015). Floor and ceiling effects in the OHS: An analysis of the NHS PROMs data set. BMJ Open.

[29] Terwee C.B., Bot S.D., de Boer M.R., Van der Windt D.A., Knol D.L., Dekker J., Bouter L.M., de Vet H.C. (2007). Quality criteria were proposed for measurement properties of health status questionnaires. J. Clin. Epidemiol..

[30] Barbero I., Vila E., Holgado F. (2011). Basic Introduction to Factor Analysis.

[31] Coe R., Merino-Soto C. (2003). Effect Size: A Guide for Researchers and Users. J. Psychol. Pontif. Catholic..

[32] Alonso J. (2003). Spanish Version of SF-36v2TM Health Survey©.

[33] Arumugam B.E.S., Nagalingam S. (2018). Derivation of Cut-Off Value for a 10-Item Opinion-Based Ordinal Survey Questionnaire. Int. J. Commun. Med. Public Health.

[34] Barua A., Kademane K., Das B., Gubbiyappa K.S., Verma R.K., Al-Dubai S.A. (2014). A Tool for Decision-Making in Norm-Referenced Survey Questionnaires with Items of Ordinal Variables. Int. J. Collab. Res. Intern. Med. Public Health.

[35] Manzi J., García M.R., Taut S. (2019). Validity of Educational Evaluations in Chile and Latin America.

[36] Byrne B. (2016). Structural Equation Modeling with AMOS: Basic Concepts, Applications, and Programming.

[37] Cheung G.W., Rensvold R.B. (2002). Evaluating Goodness-of-Fit Indexes for Testing Measurement Invariance. Struct. Equ. Model..

[38] Browne M.W., Cudeck R. (1992). Alternative ways of assessing model fit. Sociol. Methods Res..

[39] Hu L., Bentler P.M. (1999). Cutoff criteria for fit indices in covariance structure analysis: Conventional criteria versus new alternatives. Struct. Equ. Model..

[40] Bentler P.M. (1990). Comparative fit indexes in structural models. Psychol. Bull..

[41] West S.G., Taylor A.B., Wei W., Hoyle R.H. (2012). Model fit and model selection in structural equation modeling. Handbook of Structural Equation Modeling.

[42] Gaskin J. (2012). Chi Square Difference Testing. Gaskination’s StatWiki. http://statwiki.gaskination.com/index.php/CFA.

[43] Hair J.F., Ringle C.M., Sarstedt M. (2011). PLS-SEM: Indeed, a silver bullet. J. Mark. Theory Pract..

[44] Hulland J. (1999). Use of partial least squares (PLS) in strategic management research: A review of four recent studies. Strateg. Manag. J..

[45] Nunnally J.C. (1978). Psychometric Theory.

[46] George D., Mallery P. (2013). SPSS for Windows Step by Step: A Simple Guide and Reference, 11.0 Update.

[47] Halberstadt S.M., Schmitz K.H., Sammel M.D. (2012). A joint latent variable model approach to item reduction and validation. Biostatistics.

[48] Lance C.E., Butts M.M., Michels L.C. (2006). The Sources of Four Commonly Reported Cutoff Criteria. What Did They Really Say?. Organ. Res. Method.

[49] Casado-Mejía R., Botello-Hermosa A. (2018). Women’s Health. Gender and Health.

[50] Casanova M.P., Nelson M.C., Pickering M.A., Appleby K.M., Grindley E.J., Larkins L.W., Baker R.T. (2021). Measuring psychological pain: Psychometric analysis of the Orbach and Mikulincer Mental Pain Scale. Meas. Instrum. Soc. Sci..

[51] Marván M.L., Cortés-Iniestra S., González R. (2005). Beliefs about and Attitudes toward Menstruation among Young and Middle-Aged Mexicans. Sex Roles.

