# Supplementary material for: Design and Validation of a Scale to Measure Perceptions and Misconceptions about Menstruation in Nursing College Students: The METCON© Scale
Source: Healthcare (Basel). 2024 Sep 13;12(18):1836. doi: 10.3390/healthcare12181836 (PMC11431834; doi:10.3390/healthcare12181836)
Supplement: Supplementary file 1 [file healthcare-3135601-supplementary.pdf › healthcare-3135601-supplementary.pdf]

File S1. *METCON© Instrument*

Gender: Man □ Woman □ Others…….. Age: ………. years **Course:** ………………**Degree:………..**

College and University: …………………………..

Occupation: Student□ Student and Worker□

Religion: Catholic □ Protestant□ Anglican □Muslim□……Other□

I was born in: :…………………………(Town and City). **I live in**…………………………

Nacionality: ………………………………….

Please mark your answers with an X where appropriate, taking into account that:

1	2	3	4	5	NA
**Totally disagree**	**In disagreement**	**Neither agree nor disagree**	**In agreement**	**Totally agree**	**Do not answer**

	1	2	3	4	5	NA
1. At school/institute, menstruation was a more taboo subject than at home.						
2. Among friends, cousins or sisters, menstruation was a less taboo topic than at home.						
3. Among friends, cousins or sisters, menstruation was a less taboo topic than at school/institute.						
4.When I first had my period I knew what it was and I felt safe.						
5. I was scared when I first saw menstrual blood.						
6. During menstruation you feel sick or sick.						
7. Menopause means the woman ages because she no longer has menstruation.						
8. When you reach menopause, you lose sexual attractiveness.						
9. During menopause medical treatment is required (anxiety, depression, hot flashes...).						
10. Washing your head during menstruation can be dangerous for your health.						
11. If I prepare foods like mayonnaise during menstruation, it spoils or cuts easily.						
12. During menstruation you should not bathe in the sea or swimming pools.						
13. Menstrual blood is toxic, so it can have harmful effects on foods or drinks that are touched during menstruation.						
14. Menstrual blood is an accumulation of dirt and waste.						
15. Menstrual blood has an unpleasant decomposing odor.						
16. Menstrual blood is incompatible with water.						
17. During menstruation you should not have sexual relations.						
18. During menstruation you should not shower because you cut yourself and it is dangerous for your health.						
19. There were people around me (family, friends, at school) who told me what menstruation was.						
20. I know my menstrual cycle and what happens in my body.						
21. I know when in the menstrual cycle I am most fertile.						
22. I need to receive more information about the menstrual cycle.						
